# Trends for Diarrhea Morbidity in the Jasikan District of Ghana: Estimates from District Level Diarrhea Surveillance Data, 2012–2016

**DOI:** 10.1155/2018/4863607

**Published:** 2018-10-09

**Authors:** John Tetteh, Wisdom Kwami Takramah, Martin Amogre Ayanore, Augustine Adoliba Ayanore, Elijah Bisung, Josiah Alamu

**Affiliations:** ^1^Department of Epidemiology and Biostatistics, School of Public Health, University of Health and Allied Sciences, Hohoe, Ghana; ^2^Centre for Health Policy Advocacy Innovation & Research in Africa (CHPAIR-Africa), Accra, Ghana; ^3^Department of Family and Community Health, School of Public Health, University of Health and Allied Sciences, Hohoe, Ghana; ^4^Department of Epidemiology and Disease Control, School of Public Health, University of Ghana, Legon, Ghana; ^5^School of Kinesiology and Health Studies, Queen's University, Kingston, Ontario, Canada; ^6^Public Health Department, University of Illinois, Springfield, IL, USA

## Abstract

About 22% of childhood deaths in developing countries are attributable to diarrhea. In poor resource settings, diarrhea morbidities are correlated with poverty and socio-contextual factors. Diarrhea rates in Ghana are reported to be high, with cases estimated at 113,786 among children under-five years in 2011. This study analyzed the trends of diarrhea morbidity outcomes in the Jasikan District of Ghana. A retrospective analysis of records on diarrhea data for a five years' period (January 2012 to December 2016) was undertaken. There was a total of 17740 diarrhea case reports extracted from District Health Information Management System (DHIMS) II database in an Excel format which was then exported to Stata version 14 for data cleaning, verification, and analysis. Excel version 2016 was used to plot the actual observed cases by years to assess trends and seasonality. There was a period incidence rate of 272.02 per 1000 persons with a decreasing annual growth rate of 1.85%. Declines for diarrhea generally occurred from November to December and increased from January upwards, evidence that most cases of diarrhea in this study were reported in the harmattan season. High incidence of diarrhea was found to be common among under-five children and among females. Decreasing trend of diarrhea incidence which was identified in this research within the five years' period understudied shows that, by the year 2020, there will be a sharp decline in the incidence rate of diarrhea reported cases in Jasikan District, given improvements in the external environmental conditions in the district, all things being equal.

## 1. Introduction

Globally, diarrhea was a leading cause of death in all age groups (an estimated 1·31 million deaths) and a leading cause of Disability Life Years (estimated 71·59 million DALYs) among young children in 2015 [1]. The burden of deaths is greatest in low-income countries where access to water and sanitation related services is poor [1, 2]. About 22% of childhood deaths in developing countries are attributable to diarrhea [3]. In Sub-Saharan Africa (SSA), diarrhea morbidity outcomes are correlated with poverty and other sociodemographic factors [4, 5]. Diarrhea transmission routes include the contamination by the host: food or water of viruses, bacteria, or parasites [5]. Ensuring safe environmental sanitary conditions, access to clean water including handwashing and safe disposal of human waste is vital for breaking diarrhea transmission routes. Across SSA, socioeconomic factors and their influence in care seeking for diarrhea are established [4, 6, 7].

Diarrhea rates in Ghana are reported to be high. It is estimated that 113,786 cases of diarrhea were recorded in Ghana for children under-five years in 2011. Approximately 2,318 diarrhea cases reported had severe dehydration with 354 deaths within the 2011 year period [8]. Contaminated food and water are major sources for the transmission of diarrhea disease agents and also contribute to the spread of epidemics [9]. In an epidemic, the source of the contamination is usually the feces of an infected person that contaminates water and/or food. Diarrhea is transmissible and the disease can spread rapidly in areas with inadequate treatment of sewage and drinking water [10]. It has been established that the risk factors for diarrhea occurrences varied from one country to another; nevertheless the main risk factors among children included the child's age, size of the child at birth, the quality of the main floor material, mother's education and her occupation, type of toilet, and place of residence [11]. Diarrhea in Ghana is commonly caused by infectious organisms, including viruses, bacteria, protozoa, and helminths, which are transmitted from the stool of one individual to the mouth of another, termed as fecal-oral transmission. Some are well known, others are recently discovered or emerging new agents, and presumably many remain to be identified. They differ in the route from the stool to the mouth and in the number of organisms needed to cause infection and illness [12].

In the year 2017, on the occasion of Global Handwashing Day celebrated by UNICEF in every year on 15th October in Ghana, a report was shared which indicated that children under-five dying from diarrhea infections are linked to poor access to safe drinking water and sanitation which has been estimated at a rate of more than 800 per day infections [13]. It has been proven that many of these deaths could be prevented through handwashing with soap which alone can reduce diarrhea by up to 50%, yet only 20% of Ghanaians wash their hands with soap [13]. The practice of handwashing remains low in Ghana with a growth rate of about 8% since 2014 which is not encouraging; however, research proves that when children wash their hands with soap after visiting toilet and/or before eating, they reduce their risk of getting diarrhea by more than 40% [12–14]. Proper handwashing practice also contributes to the healthy development of children by keeping them in school which improves school attendance by reducing the spread of preventable diseases [13].

A number of studies in Ghana have examined diarrhea trends and morbidity outcomes in relation to environmental, pediatric rotavirus, and behavioral factors [15–18]. Regional and district level studies on diarrhea trends and morbidity are also reported in Ghana [8, 16, 19–21]. In a study conducted in the Atwima Nwabiagya District of Ghana, a total of 51,131 cases of diarrhea were reported with the episode of diarrhea greatest among children under-five between 2009 and 2013, 55.2% being females over the five years' period, and it was also found that diarrhea peaks to the highest level during the wet season in Ghana from 1995 to 2010 [21].

In the Volta Region of Ghana, Cha et al. [22] found that water supply influenced diarrhea morbidity outcomes among under-five children. In the Jasikan District of the Volta Region of Ghana where this study was conducted, district-level data show that diarrhea is among the top ten morbidity conditions at facility level from out-patient records [23, 24]. In 2015, District Health Information Management System II (DHIMS II) reported that the number of diarrhea cases in the Jasikan District was 3107, a decline from 3222 cases reported in 2011, with higher diarrhea morbidity among females compared to males between 2011 and 2015. However, there is no published study that has examined trends for diarrhea morbidity in the Jasikan District in the Volta Region of Ghana over a five years' period; hence there is no evidence of diarrhea reported cases in the district to enhance decision-making on diarrhea outcomes. This study aims to contribute to providing evidence of diarrhea morbidity trends in the Jasikan District in the Volta Region of Ghana. Routine facility-level data collected across facilities in the District for surveillance and disease tracking purposes was analyzed in this study.

## 2. Materials and Methods

### 2.1. Study Area/Setting

#### 2.1.1. Geography

The study was conducted in Jasikan District. The District was established in 1989 by Legislative Instrument (LI 1464) and is located in the northern part of the Volta Region. It shares a boundary with the Kadjebi District to the North, the Biakoye District in the West, the Hohoe Municipality in the South, and the Republic of Togo to the East. The District covers a total land area of 555.8 square kilometers representing 6.6 percent of the entire land area of the Volta Region [24].

Jasikan, the District capital, lies 110 kilometres (km) Northeast of Ho, the Regional capital and lies 260 km Northeast of Accra, the National capital. It is strategically located as it provides a good linkage between the Southeastern part of the country to the Northern Region. More importantly, the District provides a warm welcome to friends and visitors to the District as well as those passing through to the Kadjebi, Nkwanta-North, Nkwanta-South, Krachi East, Krachi-West Districts, and into the Northern Region [24].

#### 2.1.2. Disease Profile

About 59181 individuals lived in the Jasikan District, according to the 2010 Population and Housing Census where males constitute 49.2 percent and females represent 50.8 percent. The District disease profile data show that 10.9% of all household's deaths in the District were due to accident/violence or homicide while 89.1 % of deaths are due to other causes like malaria, hypertension, pneumonia, gastroenteritis, etc. The total fertility rate in the District is estimated at 3.5 for reproductive age women from 15 to 49 years [24].

#### 2.1.3. Health Systems

In terms of health systems in the District, it has reasonable health infrastructure which includes one hospital, six health centers, three community health planning services (CHPS compound), and a private clinic which are fairly distributed geographically across the District [24].

### 2.2. Study Design/Population

The study is a retrospective assessment of reported diarrhea cases from facility-level data and the study population included patient records of reported diarrhea cases in the study area from 2012 to 2016.

### 2.3. Data Source/Area

District level surveillance data from the Jasikan District (Figure 8) was extracted from the District Health Information Management System (DHIMS) II for the years 2012-2016. DHIMS is a routine facility level database that provides monthly reporting of the burden of disease morbidity across districts in Ghana under the Ghana Health Service system of data reporting. In retrieving data from the DHIMS for analysis, age and sex variables were retrieved for analysis.

For the retrieval of the data, a checklist was used to collect data from the DHIMS II database. Age groups and sex reported cases of diarrhea were extracted. The checklist was designed to collect the required data from the Jasikan District DHIMS II database.

The data was collected by

1. gathering of documents on diarrhea cases from 2012 to 2016 in the District
2. variables on age group and sex reported cases of diarrhea and date of records being recorded using the checklist
3. the data on these variables being then entered into Excel version 2016 and exported to Stata version 14 for data cleaning, verification, and analysis

### 2.4. Included and Excluded Data Cases

The study included all diarrhea cases in the District which were entered into the DHIMS II database. Cholera cases were excluded.

### 2.5. Data Processing and Cleaning

The criterion sampling method was used to draw all diarrhea cases from 2012 to 2016 from the DHIMS II database. The logic of criterion sampling was to review and study all cases that meet some predetermined criterion of importance. The method was applied by picking all diarrhea cases pertaining to the specified period under study. A checklist was used to collect data from the DHIMS II database. Patient age and sex were the two sociodemographic variables assessed and analyzed in the study. The checklist was designed to collect the required data from the Jasikan District DHIMS II database.

### 2.6. Data Analysis

DHIMS II data extracted on a checklist was entered into Excel version 2016 and then exported to Stata version 14 for data cleaning, verification, and analysis. Standard age and sex groupings for reporting DHIMS II data were used to extract data. Autocorrelation in Excel version 2016 was used to plot the actual observed cases of diarrhea by years to assess trends, seasonality, and time series of forecasting. Prediction on future diarrhea morbidity pattern was done using Stata version 14.

Age groups and sex were the two variables considered in the study. From the DHIMS II database, age was coded into 12 classes which include under 28 days, 1-11 months, 1-4, 5-9, 10-14, 15-17, 18-19, 20-34, 35-49, 50-59, 60-69, and 70 and above years for the same sex differences which in our research were recorded into under 5, 5-14, 15-19, 20-34, 35-59, and 60 and above years for the same sex differences. This category was to highlight vulnerable age groups for under-five children whose diarrhea diseases are most frequent and severe, younger children, teenagers, working age, and the retirement age in Ghana whom diarrhea diseases can also affect. We conducted a descriptive statistical analysis of the data by time, age, and person. No inferential statistics were performed. Sex estimation of diarrhea cases was done with a 95% confidence interval. The population at risk for the period under study, 2012, 2013, 2014, 2015, and 2016, comprising 62176, 63731, 65326, 66958, and 68632, respectively, was used to calculate the incidence rate for each year under study. The population at risk was estimated by the Ghana Statistical Service at the district which the Jasikan District Health Directorate uses for their estimations of disease burden. Stata version 14 was used to predict futures values of diarrhea cases from 2017 to 2020 in the Jasikan District in Ghana.

The estimated incidence rate (IR) was calculated using the formulae outlined by the Centre for Disease Control [25] as

(1) $$\begin{array}{c} \frac{\text{Number of cases}}{\text{Number of persons in the population at risk}}*1000 \end{array}$$

Growth rate estimation was calculated using the formula below [26]

(2) $$\begin{array}{c} r=\left(\;\sqrt[{n}]{\frac{P_{1}}{P_{0}}}\;\right)-1 \end{array}$$

Where r = growth rate, *P*_1_ = present incidence diarrhea cases, and *P*_0_= past incidence of diarrhea cases. Therefore,

(3) r=341537505−1r=−1.85

### 2.7. Ethical Issues

Ethical approval was sought from the Ghana Health Service Ethics Review Committee (Approval ID No: GHS-ERC 80/10/16) and permission was also obtained from the Jasikan District Health Directorate. Confidentiality was strictly observed with no patient information linked for any future identification of individual records at facility levels not being extracted.

## 3. Results

Table 1 shows demographic characteristics of diarrhea cases in Jasikan District from 2012 to 2016. There was a total period incidence of 17740 diarrhea cases with females having the highest incidence of 9758 (55.0%) reported cases. Children under-five also experienced the highest incidence within the age groupings with 9556 (53.9%) reported cases.

### 3.1. Incidence and Monthly Trends for Reported Diarrhea Cases in Jasikan District

**Figure 1:**
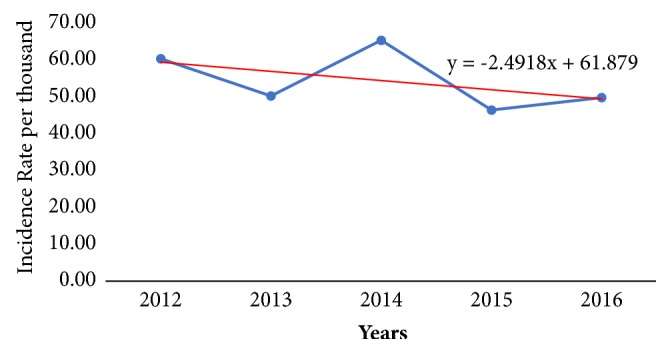
Incidence rate of diarrhea cases in Jasikan District from 2012 to 2016.

Table 2 presents findings on the annual incidence rate of diarrhea in the Jasikan District from 2012 to 2016. Between 2012 and 2016, a total of 17,740 cases of diarrhea were reported, with an estimated incidence rate of approximately 272 per thousand persons. The drop rate of diarrhea incidence within the period for the study decreased at a rate of 1.85%. The annual incidence rates range from approximately 46 to 65 cases per thousand persons within the period under study. The highest incidence rate (65.33 per thousand persons) was recorded in 2014 and the lowest incidence rate (46. 40 per thousand persons) was recorded in 2015. The pattern of incidence rate in the district within the period under study was observed to decline from 2012 to 2013 and peak from 2013 to 2014. An incidence rate of 65.33 per thousand persons is observed in 2014. A sharp decline occurred from 2014 to 2015, with a steady rise in the incidence rate from 2015 to 2016. The incidence level in 2016 was 49.76 per thousand persons (see Figure 1).

**Figure 2:**
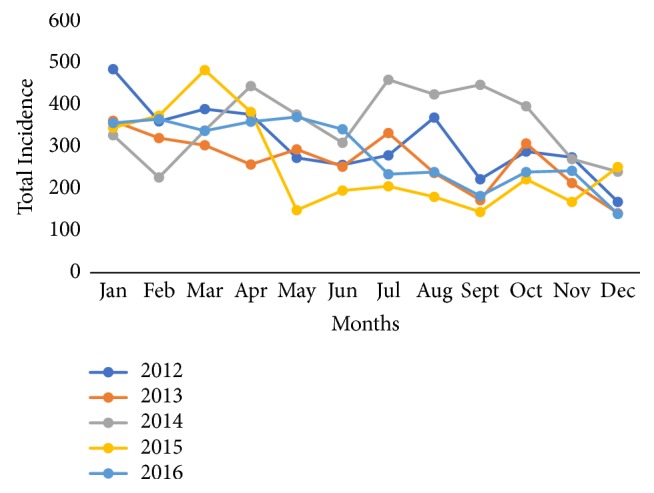
Yearly monthly patterns of diarrhea incidence in Jasikan District from 2012 to 2016.

A diagnosis of monthly trends for diarrhea morbidity shows that January 2012 recorded the highest incidence for diarrhea while December months recorded the lowest incidence rate as presented (see Figure 2). Across all years, 3 years' periods, January 2012 (485 per thousand persons), March 2012 (483 per thousand persons), and July 2014 (460 per thousand persons), recorded the highest incidence of diarrhea morbidity in the district. Monthly declines in diarrhea were lowest in December 2013 and 2016 within the study period. Although the period December was observed as a month that records a low incidence of diarrhea, the rate of decline is not consistent over the years under study. For example, there was an increase in diarrhea incidence from November 2015 (169 per thousand persons) to December 2015 (252 per thousand persons) in the district as presented in Figure 2.

### 3.2. Age and Sex Differentials and the Incidence of Reported Diarrhea Cases in Jasikan District

**Figure 3:**
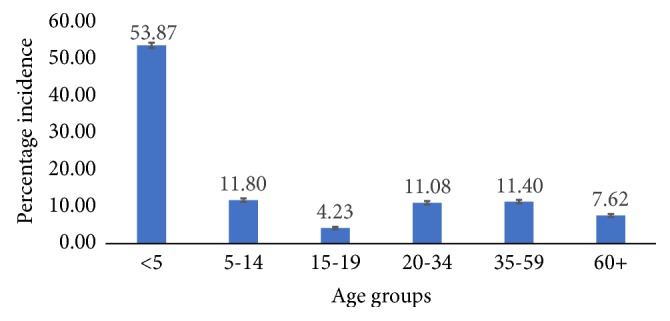
Percent incidence of diarrhea case in Jasikan District showing 95% confidence interval from 2012 to 2016 among age groups.

Age and sex categories were used to estimate the incidence of diarrhea for the various age groupings. Diarrhea cases among children under-five were the highest compared to age groupings. Diarrhea among under-five children within the period under study accounted for 53.87% which is more than half of the total number of diarrhea cases (17740) within the period under study. Records for ages between 5 and 14 years accounted for the second highest with a percentage incidence of 11.8% whiles 15-19 age group accounted for the least among the six age groupings with an incidence percentage of 4.23%. The working age accounted for 11.08% and 11.40% incidence for 20-34 and 35-59 categories, respectively. Moreover, the age group 60 years and above (retirement age) accounted for 7.62 percentage incidence of reported cases. There is a statistically significant difference of diarrhea cases in the Jasikan District among the age groupings (see Figure 3).

Table 3 shows the population incidence rate of diarrhea cases for both sexes and sex differential among children under-five in the Jasikan District. The highest incidence rate for both sexes of children under-five occurred in 2012 with an incidence rate of 235.85 per thousand persons. In terms of sex difference, the highest incidence rate occurred in 2012 among children under-five with an incidence rate of 251.71 and 220.16 per thousand persons, respectively, for males and females whereas the least occurred in 2015 and 2016 for males and females, respectively, with an incidence rate of 174.82 and 148.13 per thousand persons, respectively.

**Figure 4:**
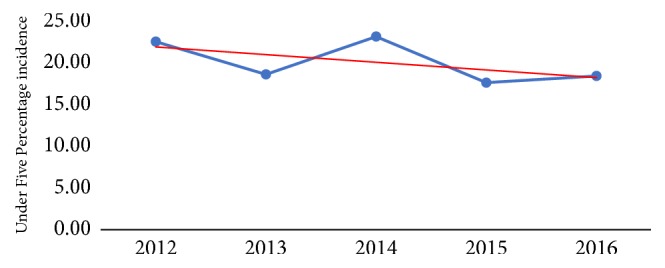
Under-five percentage incidence of diarrhea in Jasikan District (2012-2016).

In Figure 4, the pattern of under-five percentage incidence is observed to decline slowly starting from 2012, and peaking in the year 2014. A further sharp decline is observed in 2014-2015. From 2015 to 2016, a slow decline in under-five percentage incidence for diarrhea is observed. The incidence for diarrhea cases is higher in females (approximately 150 per thousand persons) compared to males (122 per thousand persons).

**Figure 5:**
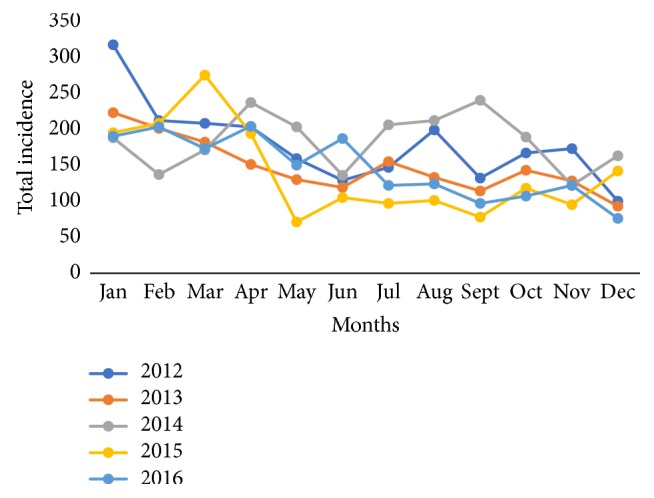
Yearly monthly patterns of diarrhea incidence among children under-five in Jasikan District from 2012 to 2016.

Monthly trends for diarrhea morbidity show January 2012 recorded the highest incidence for diarrhea among children under-five while in May 2015 recorded the lowest incidence (see Figure 5). Across all years, there are a decrease and increasing trend of diarrhea cases among children under-five from August to September and September to November, respectively, for 4 years except in 2014 which shows a wayward trend. Monthly declines in diarrhea were steady for 2012 and 2013 from January to June. Even though December is observed as a month that records low incidence of diarrhea among children under five, the rate of decline is not consistent over the years under study. It was observed that there was an increase in diarrhea incidence from November 2014 and 2015 (122 and 95 per thousand persons, respectively) to December 2014 and 2015 (163 and 142 per thousand persons, respectively) in the district (see Figure 5).

**Figure 6:**
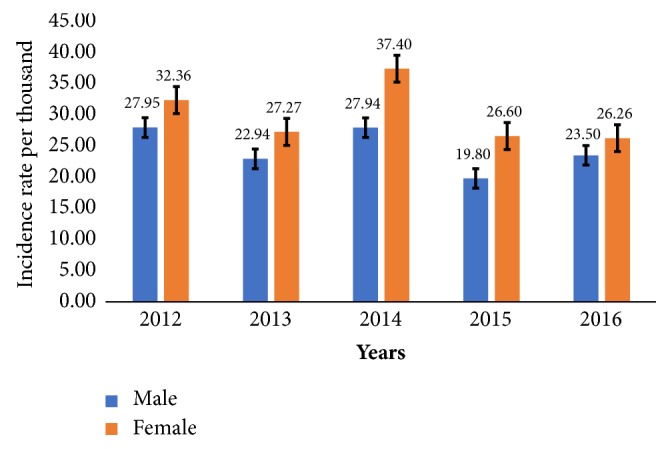
Yearly sex incidence rate of diarrhea cases in Jasikan District showing 95% confidence interval from 2012 to 2016.

The highest incidence rate (approximate figure of 30 incidences per thousand persons) for males occurred in the year 2012 while the least incidence rate (20 per thousand persons) occurred in 2015. The highest incidence rate (37 incidences per thousand persons) for females occurred in 2014 while the least incidence rate (26 per thousand persons) occurred in 2016. There is a statistical sex difference in diarrhea cases in 2014 and 2015 whereas there is no clear statistical sex difference in diarrhea cases in 2012, 2013, and 2016 (see Figure 6).

Regarding sex differentials and reported diarrhea cases, a similar trend pattern within the same years (2012-2016) is observed among males and females. In 2014, females recorded a 9% high incidence rate differential compared to males. Overall, females were observed across all years to have the highest incidence rate for reported diarrhea cases in the district (see Figure 6).

### 3.3. Seasonal Variations of Diarrhea in the Jasikan District

**Figure 7:**
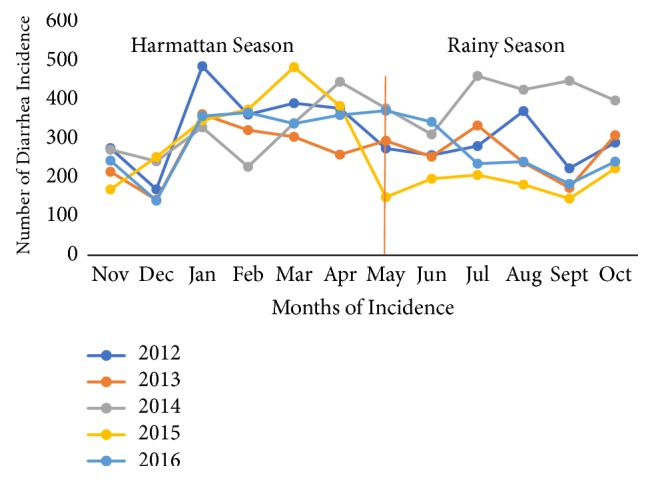
Yearly seasonal trend of diarrhea incidence in Jasikan District (2012-2016).

**Figure 8:**
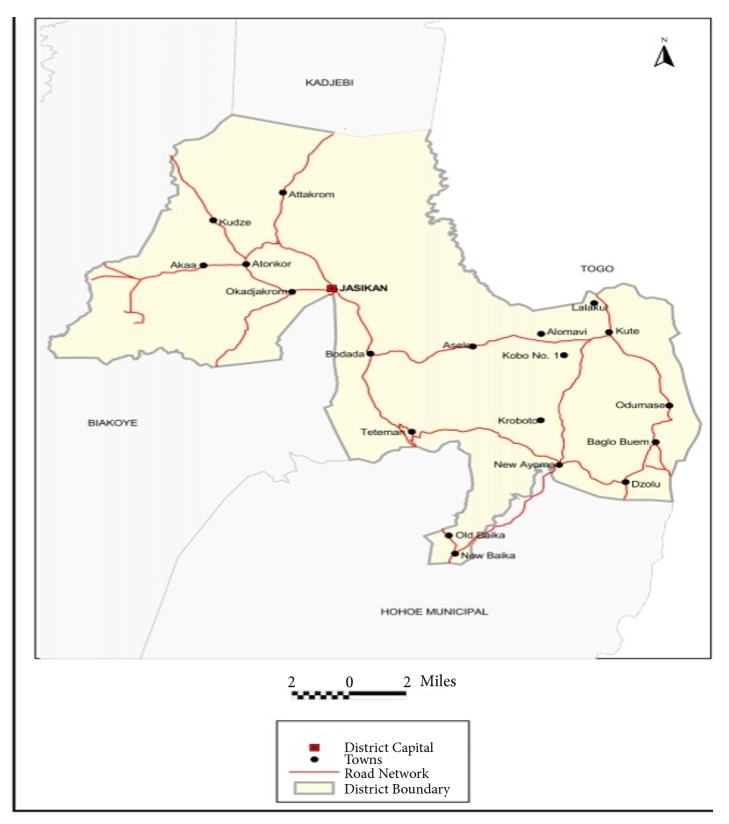
Map of Jasikan District. Source: GSS [24].

In 4 years out of the five-year period under study, diarrhea cases were highest in the harmattan season as presented in Figure 7. In aggregating all cases of diarrhea, a total of 9320 cases of diarrhea were reported in the harmattan season out of the 17,740 total reported cases of diarrhea from 2012 to 2016. The highest rainy season was observed to have more reported cases in July for 2014 compared to the harmattan season which was observed to have the highest reported in January for 2012 (see Figure 7).

## 4. Discussion

This study examined trends for reported diarrhea morbidities at facility levels in the Jasikan District of the Volta Region in Ghana using DHIMS II database system. A total of 17740 cases of diarrhea were reported in the district from January 2012 to December 2016 and a declining rate of 1.85% within the period under study was observed. The incidence of diarrhea from 2012 to 2016 was high among under-five children. The incidence of diarrhea observed here is however low compared with another 5-year diarrhea trend in the Atwima Nwabiagya District in Ghana from 2009 to 2013 [21]. Childhood diarrhea cases show a continual declining trend for all years in this study. The high incidence of childhood diarrhea found in this study corroborates district-level studies in Ghana [8, 21] and other settings in rural Kenya and globally [1, 27]. Multiple opportunistic infections for diarrhea are exacerbated in poor environmental conditions [1]. These include lack of access to clean water and inadequate personal hygiene [3].

Females had the highest levels of diarrhea incidence across all years. Overall, diarrhea incidence in this study fluctuates, with decreasing and increasing trends. A decline in diarrhea incidence was observed from 2012 to 2013, followed by a sharp increase from 2013 to 2014. In the year 2014, the months from June to November recorded higher incident levels for diarrhea. Declines in diarrhea occurred between November and December every year except in 2015. Other studies have evidenced these decreasing and increasing diarrhea trends [21, 27].

Seasonal variations in diarrhea outcome were observed with declines from November to December followed by a rise in the incidence of diarrhea from January onwards over the five-year period under study. Seasonal variations for diarrhea have been reported in Ghana [8]. In assessing yearly and monthly fluctuations, the highest incidence of diarrhea occurred in the year 2012 in the month of January. A six-month review of diarrhea morbidity in Accra, however, found April as the month that recorded the highest incidence for reported diarrhea cases [28]. Diarrhea cases were also found in this study to be high mostly in harmattan compared to the rainy season for 4-year trends, except in 2014. This differs from Anyorikeya et al. [8] that found the rainy season (May-August) as the period with a high incidence of diarrhea [8]. The difference of this research work to that of Dzotsi et al. [28] and Anyorikeya et al. [8] may be as a result of regional patterns with a different culture and different environmental conditions. Waterborne diseases increase in rainfall and decrease during drought events (harmattan season in Ghana) but in our study, we identified a higher incidence of cases in the harmattan season in Ghana which could be a result of weak monitoring system. Studies suggested that future climate change could aggravate a number of current health problems including diarrhea where changes in temperature due to global climate change can increase diarrhea disease incidence [29].

Diarrhea disease burden in Ghana is overwhelming especially among under-five children where current report estimated that over 300,000 children under-five died from diarrhea diseases which are linked to limited access to safe water, sanitation, and hygiene but only 15% of Ghanaians have access to improved sanitation [13, 14, 30].

Diarrhea disease has no discrimination in attacking an individual and anyone can catch infectious diarrhea. Our research revealed that children under five years alone accounted for more than half (53.87%) of the burden of diarrhea disease within the period under study in the Jasikan District as Figure 3 presents. A research conducted by New Hampshire Dept of Health and Human Service identified that diarrhea can spread especially quickly among babies and young children under five years who are not toilet-trained or who may not wash their hands well after going to the bathroom/toilet. It can also easily spread to the adults taking care of them and helping them with diapering and toileting [31], where this research indicates that adults aged 35-59 were the second highest (see Figure 3).

Age and sex were observed to have different outcomes from 2012 to 2016 in the study. Other studies corroborate the effect of age and sex on diarrhea outcomes [11, 32]. Females were more susceptible to diarrhea than males in the Jasikan District, accounting for an incidence rate of 149.88 per thousand persons compared to male (incidence rate of 122.14 per thousand persons). A 9% diarrhea incidence differential between female and males was found in the year 2014. Bui in 2006 also conducted a research on the most common causes and risk factors for diarrhea among children less than five years of age admitted to Dong Anh Hospital, Hanoi, Northern Vietnam which found females with high incidence for diarrhea, and also Gupta et al. [33] on risk correlates of diarrhea in children under 5 years of age in slums of Bankura, West Bengal, identified females to be more affected with diarrhea (22.89%) than males (21.73%) whereas in the South Indian study females had higher acute diarrhea (23.8%) than males (21.4%) but it was further reported that the difference was not statistically significant [33, 34].

Females having the highest diarrhea incidence has many reasons which have been found to differ in many societies with different backgrounds. It has been found that sex difference regarding rate of diarrhea has many reasons which in turn is far from clear understanding and gender preference is an unlikely explanation to diarrhea incidence because the difference has been found in different cultures and in studies with different methodological approaches [35] with an evidence that gender variations in infectious disease like diarrhea may reflect differences in gender norms [36] wherein some regions, the nutrition of female children is neglected, restricting their access to good health [33].

Currently in Ghana, there exist donor support and funding agencies that have been put in place to support improve water and sanitation and also child health through health and nutrition, water and sanitation, education, food security, etc. [37, 38]. UNICEF Ghana in collaboration with Ghana Education Service, Red Cross/Red Crescent Climate Centre, Engagement Lab at Emerson College, Right to Play, and Ghana Red Cross in their innovative ideas have introduced a programme called* Handwashing with Ananse *which is an educational game to teach children why, how, and when to wash their hands with water and soap. It is a three-chapter story and game experience centered around Ghanaian legends character who is known as* Ananse* who stole all the knowledge about handwashing and hid it in his pockets where children have to play through three scenarios with a tricky move to* Ananse* to win the handwashing knowledge back from him [39]. These interventions are really working in reducing diarrhea cases in Ghana as our research identified a declining trend of diarrhea cases with a declining rate of 1.85% and among the children under five years.

## 5. Strength and Limitation

This study presents some potential strengths and limitations.

### 5.1. Strength

The use of the DHIMS II data file for our study is relevant to bringing out how facility-level data could be used for implementation of strategies for the control of diarrhea based on context data.

### 5.2. Limitation

A limitation, however, is a potential bias of underreporting or overreporting the number of diarrhea cases that may arise from data collection and processing of this data since some facilities may lack well-trained data experts to reduce validity and reliability of data collected as this study uses only passive surveillance system dataset. DHIMS II outpatient monthly morbidity report does not currently cover all individual and household characteristics that can be used to draw associations with diarrhea and another disease morbidity at the district level. Including more individual level and household background characteristics will help estimate factors that predict the occurrence of diarrhea based on sociodemographic factors.

## 6. Conclusion and Recommendations

Evidence from our research shows that females and children under five years remain at risk of reported diarrhea cases in the Jasikan District in Ghana. These findings corroborate national and global evidence of the epidemiology of diarrhea. Successful implementation of an integrated plan toward achieving a district decline target for diarrhea by the year 2020 (see Table 4) requires commitment from health care providers and stakeholders to work hand in hand. Improving water, sanitation, and hygiene practices at household levels is key for reducing the incidence and trends of reported diarrhea cases in the District. Further studies should be designed in Jasikan District to assess district and household behavior change practices needed to promote and reduce the incidence of diarrhea-related disease conditions in the District.

A study by Cha et al. in 2015 identified water supply to influence diarrhea morbidity outcomes among under-five children in Krachi West and Krachi East districts bounded by the Jasikan District in Ghana which is close to our study setting; our research is of the view that if water supply is improved, dirrhea outcomes will be reduced drastically in the study setting.

## Figures and Tables

**Table 1:** Demographic characteristics of diarrhea cases in Jasikan District from 2012-2016.

Demographic variable	Year of diarrhea incidence					Total
	2012	2013	2014	2015	2016	
	N=3750	N=3200	N=4268	N=3107	N=3415	N=17740
	N (%)	N (%)	N (%)	N (%)	N (%)	N (%)
Sex						
Male	1738(46.3)	1462(45.7)	1825(42.8)	1326(42.7)	1613(47.2)	7982(45.0)
Female	2012(53.7)	1738(54.3)	2443(57.2)	1781(57.3)	1802(52.8)	9758(55.0)
Age grouping						
<5	2146(57.2)	1772(55.4)	2204(51.6)	1679(54.0)	1755(51.4)	9556(53.9)
5-14	362(9.7)	381(11.9)	550(12.9)	342(11.0)	458(13.4)	2093(11.8)
15-19	152(4.1)	164(5.1)	193(4.5)	105(3.4)	137(4.0)	751(4.2)
20-34	408(10.9)	334(10.4)	521(12.2)	347(11.2)	355(10.4)	1965(11.1)
35-59	385(10.3)	356(11.1)	485(11.4)	414(13.3)	383(11.2)	2023(11.4)
60+	297(7.9)	193(6.0)	315(7.4)	220(7.1)	327(9.6)	1352(7.6)

**Table 2:** Diarrhea annual incidence rate in Jasikan District from 2012 to 2016.

Year	Incidence	PR	IR
2012	3750	62176	60.31
2013	3200	63731	50.21
2014	4268	65326	65.33
2015	3107	66958	46.40
2016	3415	68632	49.76
Total	17740	326823	272.0179

**Source:** data extracted from the District DHIMS II and Jasikan District Health Directorate. **Note**: PR=population at risk, IR= incidence rate per 1000.

**Table 3:** Children under-five population incidence rate of diarrhea cases by sex in the Jasikan District from 2012-2016.

Year	Both sexes			Male			Female		
	Incidence	PR	IR	Incidence	PR	IR	Incidence	PR	IR
2012	2146	9099	235.85	1139	4525	251.71	1007	4574	220.16
2013	1772	9436	187.79	979	4693	208.61	793	4744	167.16
2014	2204	9773	225.52	1158	4860	238.27	1046	4912	212.95
2015	1679	10110	166.07	879	5028	174.82	800	5082	157.42
2016	1755	10447	167.99	977	5196	188.03	778	5252	148.13

Total	9556	48865	195.56	5132	24302	211.18	4424	24563	180.11

**Source:** Data extracted from the District DHIMS II and Jasikan District Health Directorate. **NOTE:** PR=Population at Risk, IR= Incidence Rate per 1000

**Table 4:** Forecasting trend of diarrhea incidence from 2017-2020.

Year	Time	Incidence
2017	6	3319.1
2018	7	3242.8
2019	8	3166.5
2020	9	3090.2

## Data Availability

The data used to support the findings of this study are available from the corresponding author upon request.

## Abbreviations

AFRO: African Regional Office
AIDS: Acquired Immune Deficiency Syndrome
Birr: Ethiopian Currency
CDC: Centre for Disease Control
CFR: Case Fatality Rate
DRC: Democratic Republic of Congo
DHIMS: District Health Information Management System
EOF: Empirical Orthogonal Function
GHS: Ghana Health Service
HIV: Human Immunodeficiency Virus
ICCDR: International Centre for Diarrhea Disease and Research
IDSR: Integrated Disease Surveillance and Response
MoH: Ministry of Health
UNICEF: United Nations International Children's Emergency Fund
WHO: World Health Organization.
